# The Correlation of PPAR*α* Activity and Cardiomyocyte Metabolism and Structure in Idiopathic Dilated Cardiomyopathy during Heart Failure Progression

**DOI:** 10.1155/2016/7508026

**Published:** 2016-02-15

**Authors:** E. Czarnowska, D. Domal-Kwiatkowska, E. Reichman-Warmusz, J. B. Bierla, A. Sowinska, A. Ratajska, K. Goral-Radziszewska, R. Wojnicz

**Affiliations:** ^1^Department of Pathology, The Children's Memorial Health Institute, Aleja Dzieci Polskich 20, 04-730 Warsaw, Poland; ^2^Department of Biochemistry, Medical University of Silesia in Katowice, Jedności Street 8, 41-200 Sosnowiec, Poland; ^3^Department of Histology, School of Medicine with the Division of Dentistry, Medical University of Silesia in Katowice, Jordana Street 19, 41-808 Zabrze, Poland; ^4^Department of Pathology, The Medical University of Warsaw, T. Chałubińskiego Street 5, 02-004 Warsaw, Poland; ^5^Department of Genetics and Animal Breeding, Faculty of Animal Science, Warsaw University of Life Sciences-SGGW, Ciszewskiego Street 8, 02-786 Warsaw, Poland; ^6^Department of Histology and Embryology, Medical University of Silesia in Katowice, Jordana Street 19, 41-808 Zabrze, Poland

## Abstract

This study aimed to define relationship between PPAR*α* expression and metabolic-structural characteristics during HF progression in hearts with DCM phenotype. Tissue endomyocardial biopsy samples divided into three groups according to LVEF ((I) 45–50%, *n* = 10; (II) 30–40%, *n* = 15; (III) <30%, *n* = 15; and control (donor hearts, >60%, *n* = 6)) were investigated. The PPAR*α* mRNA expression in the failing hearts was low in Group (I), high in Group (II), and comparable to that of the control in Group (III). There were analogous changes in the expression of FAT/CD36 and CPT-1 mRNA in contrast to continuous overexpression of GLUT-4 mRNA and significant increase of PDK-4 mRNA in Group (II). In addition, significant structural changes of cardiomyocytes with glycogen accumulation were accompanied by increased expression of PPAR*α*. For the entire study population with HF levels of FAT/CD36 mRNA showed a strong tendency of negative correlation with LVEF. In conclusion, PPAR*α* elevated levels may be a direct cause of adverse remodeling, both metabolic and structural. Thus, there is limited time window for therapy modulating cardiac metabolism and protecting cardiomyocyte structure in failing heart.

## 1. Introduction

Heart failure (HF) can be of various etiology [1]. Despite substantial improvement in HF prevention and management, this clinical syndrome continues to be a major health concern, since more than 50% of HF patients die within 5 years of being diagnosed [2]. Therefore, an enhanced understanding of differences in cardiac status during the disease's progress is required for more precise therapy.

Routine histopathological examinations of human cardiac muscle collected* via* endomyocardial biopsy demonstrate the diversity of anomalies in myocardial tissue but have not been used to establish their peculiarities in association with metabolic changes and the degree of cardiac dysfunction. Cardiac remodeling underlying HF progression is known to be associated with cardiomyocyte hypertrophy, structural impairment and death, and fibrosis irrespective of disease etiology [3] on one hand and with changes in substrate uptake and metabolism [4] on the other. Most studies reveal reduced fatty acid (FA) use and impaired glucose oxidation in the failing heart [5]. The uptake and use of both substrates are closely linked and coregulated by the peroxisome proliferator-activated receptors alpha (PPAR*α*) that is highly expressed in the myocardium [6, 7]. Findings in PPAR*α*-null mice [8] and MHC-PPAR*α* mice [9] suggest that the lack of PPAR*α* or increase in its level leads to cardiac hypertrophy and dilated cardiomyopathy (DCM). Furthermore, experimental data show that overactivation of PPAR*α* is associated with impaired cardiac function [10–13]. The research on PPAR*α* in the failing human heart had concerned only end-stage HF [14–18].

Therefore, to enhance our understanding of the phenomena associated with HF progression and DCM phenotype we analyzed sections of endomyocardial tissue taken during biopsy sampling. The aim of this study was to define the temporal relationship between PPAR*α* expression and cardiomyocyte metabolic and structural remodeling in idiopathic DCM during HF progression.

Analyses revealed phase changes of PPAR*α* during HF, among which significant structural damage and cardiomyocyte death were associated with PPAR*α* increase.

## 2. Material and Methods

### 2.1. Patients

Forty consecutive patients with idiopathic dilated cardiomyopathy (iDCM) and six donor hearts (which had not been transplanted for technical reasons), all from the database of Silesian Medical University (Poland), were included in this study. All tissue samples were obtained with a signed consent from patients or patients' families. Exclusion criteria included diabetes, secondary heart failure (due to hypertension, ischemic heart disease, primary valvular disease, and alcohol abuse), and familial disease.

DCM groups were defined according to left ventricle ejection fraction (LVEF): Group (I) with mildly reduced LVEF of 45–50% (*n* = 10), Group (II) with moderately reduced LVEF of 30–40% (*n* = 15), and Group (III) with severely reduced LVEF of <30% (*n* = 15). Donor hearts (LVEF > 60%, *n* = 6) were served as the control group. The age of patients in groups with HF was between 30 and 40 years, and only single subjects had about 50 years, while in the control group the age was between 22 and 40 years. All patients with HF were on typical therapeutic regimens including digitalis, diuretics (furosemide, 40–80 mg/d, and spironolactone, 100 mg/d), an ACE inhibitor (captopril 50–75 mg/d), *β*-blockers (metoprolol, 50 to 100 mg/d), and an antiarrhythmic drug (amiodarone, 200 to 400 mg/d). Detailed characteristics of entire study population are presented in Table 1.

### 2.2. Histopathology with Morphometric Analyses

Deparaffinized 5 *μ*m thick sections of biopsy samples taken for diagnostic reasons were routinely stained with hematoxylin and eosin (HE), Masson's Trichrome (MT), and periodic acid Schiff (PAS) and PAS with diastase digestion and described for general histopathology. Images from these sections taken under identical lightning conditions were analyzed by two independent investigators blinded to the NYHA class and LVEF value. Measurements were done with the Cell^sense^ program (Olympus). Finally, obtained data were presented as mean ± SEM for each group. Cardiomyocyte cross-sectional area and longitudinal area were measured systematically throughout the entire tissue section. Fibrosis quantification was performed on TM stained sections with the software by defining blue stained fibrotic areas automatically as the percentage of the total analyzed tissue. PAS-positive area was calculated as the percentage of the whole analyzed tissue section. The percentage of PAS-positive scored as 0 for area 5%, as 1 for area 6–30%, as 2 for area 31–60%, and as 3 for area >60%. Additionally, PAS staining intensity was scored as 0 for lack of staining, 1 for weak positive staining, 2 for moderate positive staining, and 3 for strong staining. The final score of glycogen accumulation resulted from both PAS staining intensity and the percentage of stained area as follows: normal glycogen abundance for a score of <1, low glycogen storage for a score of 1–4, and high glycogen storage for a score of ≥5.

### 2.3. Cardiomyocyte Immunohistochemistry and Numerical Cell Density

Deparaffinized 5 *μ*m thick sections mounted on poly-L-lysine coated slides were blocked against endogenous peroxidase and nonspecific binding of the respective primary antibodies. Anti-desmin antibodies (1 : 50, DakoCytomation) and anti-ubiquitin antibodies (1 : 50, Abcam) were used for visualization of the desmin cytoskeleton and the intensity of proteasome/lysosomal degradation to estimate cardiomyocyte impairment and degeneration. Next, the secondary IgG antibody conjugated with horseradish peroxidase (EnVision System HRP ready to use, DakoCytomation) was applied. Nuclei were stained with hematoxylin. Numerical density of cardiomyocytes with pathology of the desmin cytoskeleton (according to the features described in detail previously [19]) was identified in each section and calculated as the percentage of the total cardiomyocyte number. Four categories of desmin patterns in cardiomyocytes were identified: normal, with regular distribution of fine desmin fibrils (type I), compensatory pattern, with increased desmin expression and properly arranged desmin (type IIA), and pathological pattern, with desmin visible as intercellular aggregates (type IIB) or with lack of desmin staining (type III).

Apoptotic and autophagic cardiomyocyte death was visualized with anti-active caspase-3 (1 : 200, Millipore) and anti-Beclin-1 (1 : 500, Novus Biotechnologies) primary antibodies, respectively. The secondary detection system was Alexa Fluor 488 goat anti-rabbit (Invitrogen). Nuclei were stained with DAPI (1 *μ*L/1 mL, Millipore). Slices were investigated with a confocal microscope (FV-1000, Olympus). The autophagic and apoptotic cardiomyocytes were presented as the percentage of the total cardiomyocyte number.

The specificity of all staining was verified by omission of primary antibodies. All analyses were done in a blinded manner by two independent investigators.

### 2.4. Ultrastructure and Analysis

Biopsy sections routinely stained for ultrastructural evaluation and obtained from tissue samples processed into Epon blocks were examined under an electron microscope (Jem 1011, Jeol) and morphometrically evaluated using iTEM software. The intensity of anomalies in cardiomyocytes was graded as follows: contractile apparatus with thickened or blurred Z-bands (grade 1), myofibril loss (grade 2); mitochondria of normal shape, increased in number, and forming clusters (grade 1), polymorphic mitochondria with locally lucent matrix and lost cristae and decreased in number (grade 2); T-tubule loss at the Z-line (grade 1), dilated T-tubules with absent sarcoplasmic reticulum (SR) junction (grade 2); isolated lipid droplets in the cytoplasm (grade 1), numerous lipid droplets (grade 2); dispersed glycogen with slight accumulation in the perinuclear area and between myofibrils (grade 1), abundant glycogen throughout the cytoplasm (grade 2). Cumulative intensity of ultrastructural pathologies was calculated for each patient as a mean value. Additionally, the numerical density of cardiomyocytes with ultrastructural anomalies was determined and scored as 0 for 5%, 1 for 6–30%, 2 for 31–60%, and 3 for >60%. The final ultrastructural pathology score was calculated from total points obtained in both analyses and described as no pathology for score ≤2, mild pathology for score 3, moderate pathology for score 4, and severe pathology for score 5.

### 2.5. Quantitative Real-Time PCR

Total myocardial RNA was extracted from frozen myocardial tissue sections via a standard method. The quality and quantity of RNA were determined using gel electrophoresis and the spectrophotometric method (GeneQuant II RNA/DNA Calculator (Pharmacia Biotech)). Reverse transcription, followed by quantitative polymerase chain reaction (RT-PCR) for PPAR*α*, FAT/CD36, CPT-1, GLUT4, PDK-4, Beclin-1, and caspase-3 mRNAs, was done according to a previously reported technique [20]. The mRNA copy numbers of examined genes were determined on the basis of the commercially available standard of *β*-actin (TaqMan DNA Template Reagent Kit, Applied Biosystems) and expressed per *μ*g of total RNA. The quantity of PCR amplicons was determined after each round of amplification using the fluorescent dye SYBR-Green I (Qiagen). Oligonucleotide primers are shown in Table 2.

### 2.6. Statistical Analyses

All data are presented as mean ± standard deviation. A nonparametric approach was used, since the patient population did not show a normal distribution of clinical variables (Shapiro-Wilk normality test). The Mann-Whitney, Kruskal-Wallis, and* post hoc* tests were used for the statistical significance of differences yielded by mRNA evaluations. Then, Spearman coefficients were calculated for PPAR*α* expression and metabolic and structural parameter in each investigated group and in the whole population with HF. Differences between groups were considered significant when the *p* value was <0.05.

## 3. Results

### 3.1. Expression of PPAR*α* and the Related Genes during HF Progression

**Figure 1:**
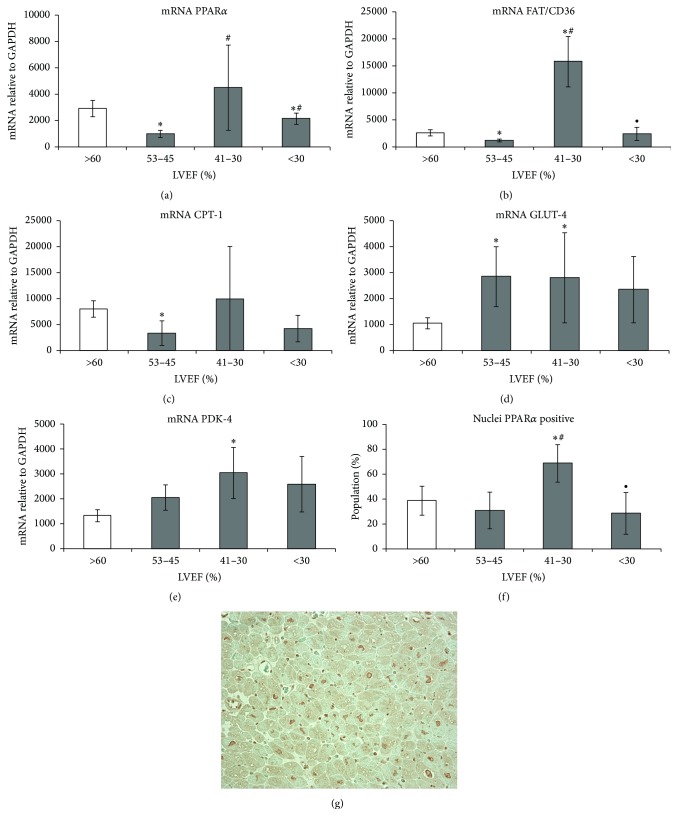
Changes in PPAR*α* activity and in the related genes involved in fatty acid and glucose metabolism in the myocardium of the human heart during heart failure progression. Myocardial expression of (a) PPAR*α* mRNA; (b) FAT/CD36 mRNA; (c) CPT-1 mRNA; (d) GLUT-4 mRNA; (e) PDK-4 mRNA. GPDH was used as a housekeeping gene. Results are presented as mean ± SEM; ^*∗*^ *p* < 0.05 versus control; ^#^ *p* < 0.05 versus Group (I); PPAR*α* expression in myocardial tissue section. (f) Morphometric analysis of cardiomyocyte population with PPAR*α*-positive nuclei; results are presented as mean ± SEM; ^*∗*^ *p* < 0.05 versus control; ^#^ *p* < 0.05 versus Group (I); ^∙^ *p* < 0.05 versus Group (II). (g) A representative image showing the expression of PPAR in the myocardial tissue section of the heart patient from Group (II).

We detected a decrease in PPAR*α* expression in hearts with mildly reduced EF (Group I), a marked increase in patients with moderate cardiac dysfunction (Group II), and a renewed decline in hearts with severely reduced EF (Group III) in comparison with the control group (Figure 1). Similar, heart failure stage-related changes were observed for FAT/CD36 and CPT-1 mRNA levels in Groups (I) and (III). However, there was only a tendency of CPT-1 mRNA level increase in Group (II) compared with the control. Subsequently, there was high expression of GLUT-4 mRNA in all failing hearts and high expression of PDK-4 mRNA in Group (II), compared with control.

In agreement with PPAR*α* gene expression were results of analysis of sections stained with anti-PPAR*α* antibody showing a significant increase of cardiomyocytes with PPAR-positive nuclei in the endomyocardial tissue from hearts with moderately reduced LVEF (Figure 1).

### 3.2. Cardiomyocyte Hypertrophy, Structural Impairment, and Myocardial Fibrosis during HF Progression

**Figure 2:**
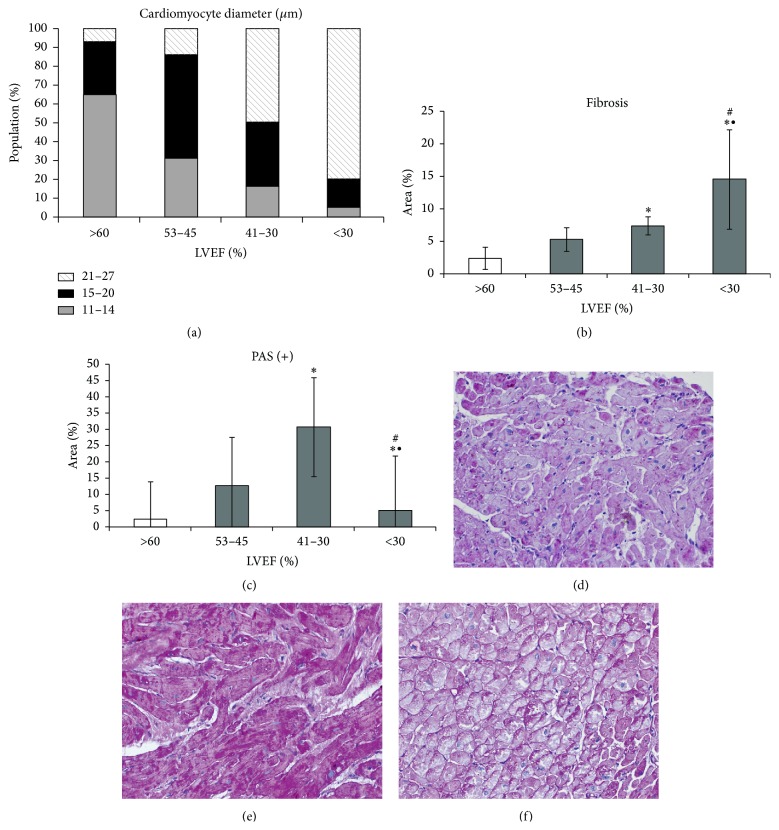
Results of morphometric analyses in tissue section: (a) cardiomyocyte hypertrophy; (b) fibrosis; (c) PAS-positive area; results as mean ± SEM; ^*∗*^ *p* < 0.05 versus control; ^#^ *p* < 0.05 versus Group (I); ^∙^ *p* < 0.05 versus Group (II); representative images of PAS-stained endomyocardial tissue sections from hearts with (d) LVEF 53–45%, (e) LVEF 41–30%, and (f) LVEF < 30%.

Cardiomyocyte hypertrophy was expressed by an increase in cell cross-sectional and longitudinal areas compared with these parameters in the control group (Table 3, Figure 2). There was a significant increase in the length of cardiomyocytes in contrast to their diameter in the early stages of heart failure, while a significant increase in cardiomyocyte diameter was found in the group with moderately reduced heart function. Cellular hypertrophy was accompanied by nuclear enlargement, loss of regular striation in the contractile apparatus in HE stained sections, and appearance of abundant PAS-positive material within the cytoplasm (Figure 2). PAS-positive staining was visible in the form of discrete diffuse pattern in the group with LVEF of 50–45%, strong staining in most cardiomyocytes in the group with LVEF of 41–30%, and renewed diffuse or irregular staining in the group with severely decreased EF (Figures 2(d)-2(e)). Morphometric analysis of PAS-positive areas showed the greatest abundance of glycogen in the heart sections in Group (II).

Fibrosis sharply increased only in end-stage HF (Figure 2). The phenomenon was characterized by fine accumulation of collagen between cardiomyocytes and fascia between groups of cells in the hearts with mildly abnormal EF; however, there was no significant difference in comparison with control. Fibrosis with focal collagen accumulation between cardiomyocytes was seen in the hearts of Group (II) and continued further with the decrease of EF, suggesting replacement of dead cells. A small increase of fibrotic tissue was also observed around blood vessels in tissue samples from the hearts with severely reduced LVEF.

**Figure 3:**
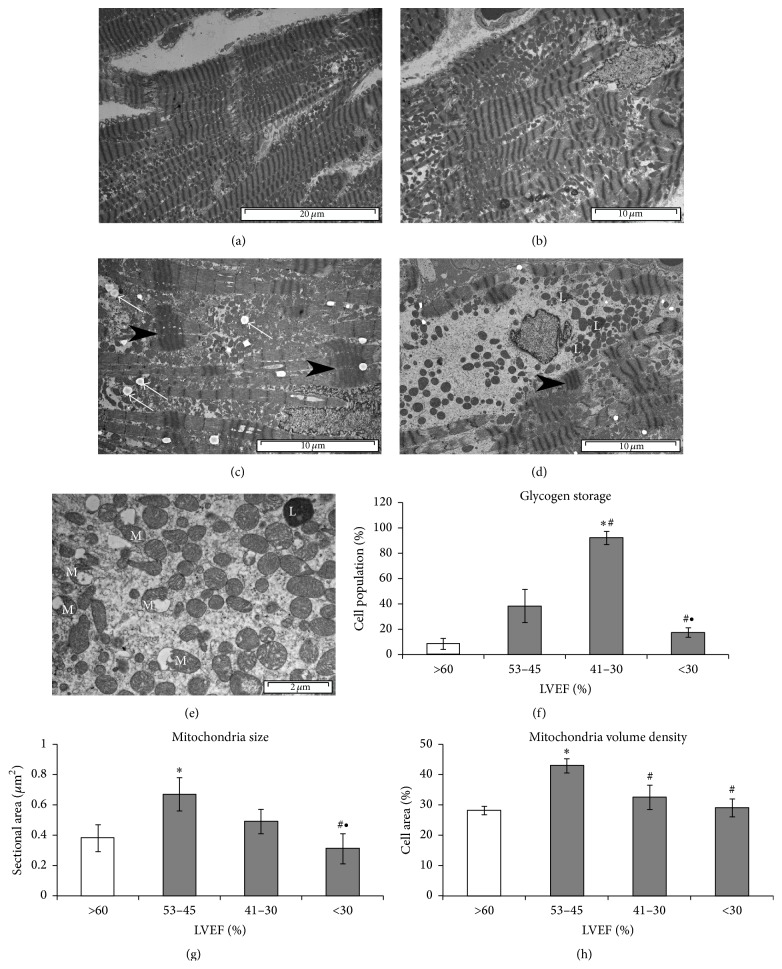
Cardiomyocyte ultrastructural remodeling during heart failure progression. Representative images for (a) control heart. Cardiomyocytes with normal ultrastructure and oval mitochondria arranged in rows. (b) Heart Group (I) (LVEF 53–45%). Cardiomyocyte with increased number of various size mitochondria arranged in clusters, partially missing contractile apparatus, incidence of dilated T-tubule. (c) Heart Group (II) (LVEF 41–30%). Cardiomyocyte with missing contractile apparatus and contraction bands (black arrowheads), numerous polymorphic mitochondria, lipid droplets between mitochondria (white arrows), and (d) Heart Group (III) (LVEF < 30%). Cardiomyocyte with severe loss of contractile fibrils and contraction band (black arrowhead), scattered mitochondria, and lipofuscin granules (L). (e) Impaired mitochondria (M) in hearts with LVER < 30%; results of morphometric analyses. (f) Glycogen storage. (g) Mitochondria size. (h) Mitochondria volume density. Results as mean ± SEM; ^*∗*^ *p* < 0.05 versus control; ^#^ *p* < 0.05 versus Group (I); ^∙^ *p* < 0.05 versus Group (II).

Changes of cardiomyocytes ultrastructure graded with HF progression (Figure 3). Mild ultrastructural changes appeared in cardiomyocytes in the hearts with mildly reduced EF. Significant increase in the number of mitochondria (about twofold increase in volume density compared with the control group) and low incidence of dilated T-tubules or a decreased T-tubule density or myofibril loss were characteristic features in these hearts. However, contraction bands were often seen. In contrast, in Group (II) irregular distribution and widening of sarcomeres and the T system, mitochondria polymorphism and varied size and damage of individual, and T-tubule-SR junction loss were accompanied by an abundance of glycogen in the cytoplasm, and numerous lipid droplets located close to mitochondria. Intensified changes, such as progressive loss of myofibrils, disorganized sarcomeres, scattered and decreased number of mitochondria, swollen mitochondria (Figure 3(d)), or loss of mitochondrial cristae and lipofuscin granules located in perinuclear area, were observed in Group (III). In this group over 30% of cardiomyocytes exhibited significant pathological changes. Important, significant differences in the accumulation of glycogen and mitochondria size and volume to the entire population of mitochondria were evident in the hearts of myocardial tissue cardiomyocytes Group (II) compared with Group (III) (Figure 3).

### 3.3. Cardiomyocyte Degeneration and Death

**Figure 4:**
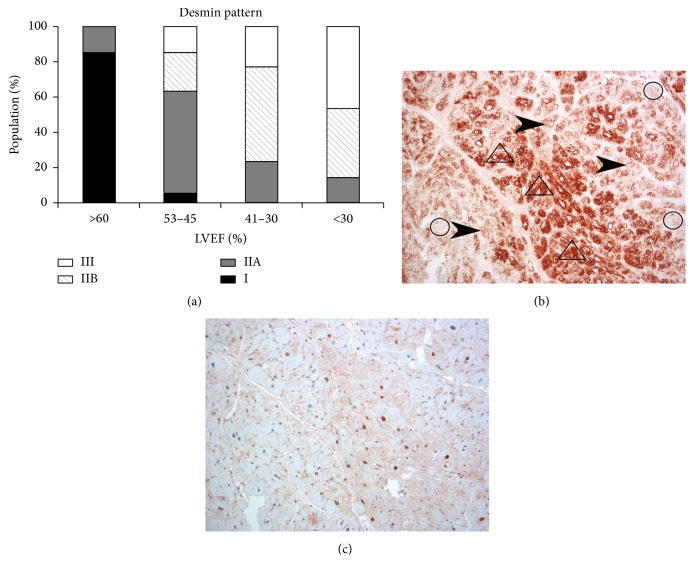
Desmin pattern in cardiomyocytes of failing heart. (a) Changes of desmin expression pattern in relation to the degree of heart failure. (b) A representative image showing the different types of desmin in the myocardium tissue of the patient's heart in Group (II): increased expression (arrowhead), desmin forming granular clusters (in triangles), or desmin disappearance (in circle). (c) Expression of ubiquitin in serial tissue section shown in (b).

Cardiomyocyte degeneration related to desmin cytoskeleton injury. Desmin cytoskeletal anomalies manifested themselves in the form of increased appearance of desmin forming properly ordered structures (compensation pattern marked as type IIA) or granular structures (pathological pattern marked as type IIB) or showing a loss of desmin (pathological pattern marked as type III) (Figures 4(a) and 4(b)). Normal (pattern marked as type I) or compensatory desmin pattern corresponded to mild ultrastructural changes in cardiomyocytes, while pathological pattern corresponded to moderate and severe ultrastructural injury. Desmin pathological pattern was visible in increasing number of cardiomyocytes with HF progression. In addition, cardiomyocytes with a pathological IIB pattern of the desmin cytoskeleton demonstrated diffuse staining for anti-ubiquitin antibodies in the cytoplasm and nuclei (Figures 4(b) and 4(c)).

There was negligible cardiomyocyte death, autophagic and apoptotic, in Group (I); however the intensity of dying increased significantly with a reduction in LVEF (Table 3).

### 3.4. Association between PPAR*α* and Metabolic and Structural Anomalies

**Figure 5:**
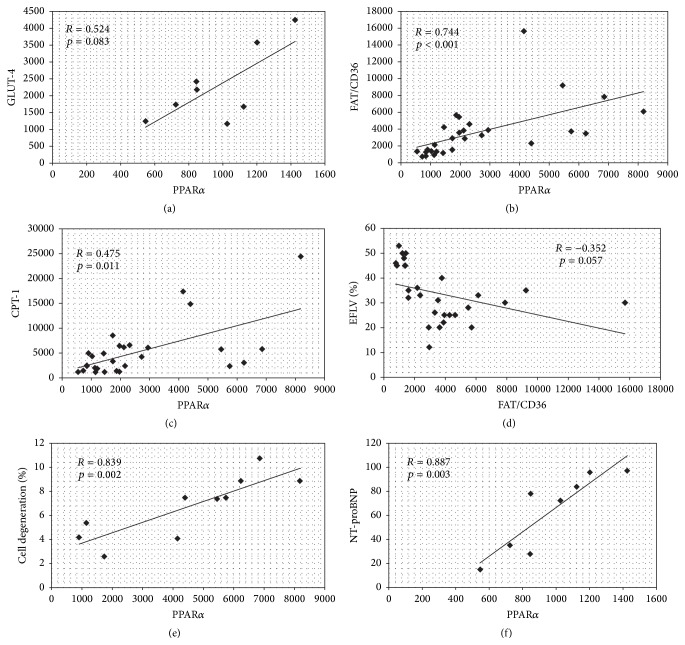
Association between PPAR*α* and metabolic and structural anomalies. Results of analyses for patients Group (I): (a) PPAR*α* and GLUT-4 mRNA for patients Group (I) and for all hearts with HF: (b) PPAR*α* and FAT/CD36 mRNA; (c) PPAR*α* and CPT; (d) FAT/CD36 mRNA and LVEF and for patients Group (II): (e) PPAR*α* mRNA and cell degeneration and (f) PPAR*α* mRNA and serum NT-proBNP level.

Positive link between mRNA PPAR*α* and GLUT-4 was observed only in Group (I), however with weak significance (*p* = 0.083) (Figure 5(a)). In contrast, we found a correlation between the expressions of PPAR*α* mRNA and FAT/CD36 and CPT-1 mRNA for the entire study population (Figures 5(b) and 5(c)). Simultaneously, there was a strong trend in negative correlation between FAT/CD36 and LVEF (Figure 5(d)). Interestingly, a significant correlation between PPAR*α* and cardiomyocytes degeneration (Figure 5(e)) and serum NT-proBNP level (Figure 5(f)) was found for tissue samples in Group (II) (Figure 5(e)), but not for whole failing heart population, suggesting link PPAR*α* overexpression and structural damage.

## 4. Discussion

In this study, we demonstrate for the first time the variability of PPAR*α* mRNA levels in association with expression of genes involved in fatty acid and glucose metabolism during HF progression. Furthermore, PPAR*α* overexpression in HF with moderately reduced LVEF suggests a maladaptive effect on cardiomyocyte structure.

Although altered PPAR*α* expression in failing human hearts had already been reported for end-stage HF and the results, even for this stage, had been inconsistent, namely, a decreased [14], increased [16, 17, 21], or unchanged PPAR*α* expression in comparison with that in the donor hearts [22] had been reported. In those studies the LVEF values were in range of these in patients from Groups (II) and (III) in our study. It should be emphasized that contradictory results as to PPAR*α* expression were also reported in experimental HF models with accompanying DCM phenotype (e.g., [21, 23–25]).

Changes in PPAR*α* expression are known to involve changes in FA and glucose metabolism, as activation of PPAR*α* in the myocardium increases the expression of genes involved in FA uptake, mitochondrial *β*-oxidation, while reduced PPAR*α* activity leads to increased glucose uptake and use [4]. In our study, the hearts with mildly reduced LVEF (Group I) pattern of expression for genes involved in cardiac metabolism suggested an increase in intensity of glucose metabolism and decline in FA metabolism before a significant cardiomyocyte enlargement and cardiac hypertrophy was observed. Apart from an increased expression of GLUT-4, we found tendency to decrease of PDK-4 mRNA level. In this context only negligible accumulation of glycogen inside cardiomyocytes suggested an effective glycolysis and/or rapid turnover of glycogen. This metabolic pattern seems to be adaptive since cardiomyocyte death was also negligible and cardiomyocytes exhibited compensatory pattern of desmin cytoskeleton and normal ultrastructure. The increased number of mitochondria was probably a result of mitochondria biogenesis activated in order to adapt to energetic requirements in conditions of stress by the means of increasing glucose metabolism [26]. This is especially probable as the decrease in mitochondrial fatty acid oxidation (FAO) has been suggested as a predictive factor of the onset of contractile dysfunction [27, 28].

Metabolic patterns in the hearts with moderately reduced LVEF characterized by increased expression of FAT/CD36 and PDK-4 mRNA concomitant with a tendency towards increased CPT-1 levels and the accumulation of glycogen and lipid droplets in the cytoplasmic pool were linked to PPAR*α* gene overactivation. It is known that PPAR*α* may modify the expression of PDK-4 which phosphorylates the pyruvate dehydrogenase inhibiting the rate of glucose oxidation; therefore the resulting excess of glucose in the internal pool is stored in the form of glycogen [29]. Accumulation of lipid droplets in cardiomyocytes can be the effect of triglyceride (TG) synthesis dependent on FA uptake [30] or a decrease in FA liberation by hydrolysis [31]. The triglyceride pool has been also identified as a signaling factor for the regulation of PPAR*α* activity [32]. Additionally, a toxic effect of lipid accumulation as well as positive correlation between lipid accumulation and cardiac dysfunction has been demonstrated in human and animal models [9, 33]. Furthermore, myocardial lipid accumulation has been demonstrated in association with mitochondrial uncoupling and ROS overproduction [34]. The observed increased FA uptake and lower glucose uptake in Group (II) hearts in our study are generally consistent with PET scan findings in human hearts with LVEF <35% [34]. Furthermore, the fact that PPAR*α* overactivation linked with cellular structure abnormalities is consistent with studies in mice MHC-PPAR*α* [9] and with the use of a PPAR*α* agonist in PPAR*α*-null mice [8]. It is known that one of the factors directly responsible for cell rebuilding process in the failing heart is the excessive production of free radicals, which is concomitant with damage to mitochondrial structure [35]. Thus, mitochondrial anomalies observed in cardiomyocytes in HF Group (II) are consistent with this observation. Additionally, glycogen storage in cardiomyocytes has been shown to be associated with cardiomyocyte apoptosis [36–38]. However our investigations not confirmed link to apoptosis but rather suggested association with autophagic death. A strong mediator of autophagy could be damaged mitochondria (demonstrated here) and mitochondria ROS overproduction (demonstrated by others). Thus cardiac FA use can be considered as unfavorable for heart at this disease stage. Additionally, PPAR*α* may be involved in facilitating some structural features responsible for cardiac function depression, particularly as we found a strong trend of negative correlation between FAT/CD36 mRNA levels and LVEF values in failing heart. Increased cardiomyocyte degeneration (revealed by severe pathology of desmin cytoskeleton in correlation with ultrastructural cell damage) in the hearts with moderately reduced LVEF might relate to the unfavorable effects of glycogen and lipid storage suggested in literature [33, 36–38] and is consistent with the findings of Hein et al. (2003) [39]. Thus, the observed increased expression of ubiquitin in cardiomyocytes with damaged desmin cytoskeleton came as no surprise.

In end-stage HF we found that PPAR*α* mRNA levels returned to the level found in the control group, contrary to other investigators who reported its either decrease [14, 15] or increase [17]. In this HF stage low fatty acid oxidation [40] and decreased glycolysis [41] have been reported. In our study, cardiomyocytes structural damage manifested by the loss of desmin cytoskeleton and ultrastructural pathology along with the accumulation of lipid droplets and loss of glycogen may correspond to those described by these authors changes in energy metabolism. Additionally, the magnitude of structural changes and cardiomyocyte death probably contributed to left ventricular dysfunction. Important, structural changes strongly associate with HF mortality and recurrence as has been reported recently by Saito et al. (2015) [42].

A potential mechanism of PPAR*α* activity reduction at the early phase of HF remains unclear. From literature data comes the fact that it can be hypoxia [43] but this unlikely was not the case in our study, since we have not found evidence of significant cardiomyocyte hypertrophy or fibrosis and vascular changes in patients with mildly reduced LVEF. Thus, the role of activation of hypertrophic pathway and stress accompanying left ventricular dilatation should be taken into consideration, while increased PPAR*α* expression during heart failure progression might be an effect of long-term adrenergic activation and the presence of lipid droplets inside cardiomyocytes, as both are characteristic for progressive HF [32, 43].

In summary the goal of this study was to show PPAR*α* alterations during HF progression in association with cardiomyocyte metabolic and structural features in patients relatively young in contrast to other investigators, although the rigorous criteria for entry to study limited the number of patients in each group. Furthermore limited amount of tissue was a cause that we could not carry out protein expression analysis for referenced genes.

In conclusion, patients with DCM and progressive HF exhibit specific cardiac metabolism related to PPAR*α* expression. This metabolism involves increased PPAR*α* levels in a narrow time window that is parallel with significant cardiomyocyte injury. The observed pattern of changes during HF progression suggests other targets than modulation of cardiac metabolism as a whole, for example, mitochondria, ROS. Therefore it is not a surprise that results of FA metabolism modulation in failing heart still remain controversial delivering contrary results, while modulation of glucose metabolism in human studies has demonstrated only short-term benefit (reviewed in [5]).

## Figures and Tables

**Table 1:** Clinical data.

	Control (*n* = 6) LVEF > 60%	Group (I) (*n* = 10) LVEF 50–45%	Group (II) (*n* = 15) LVEF 44–30%	Group (III) (*n* = 15) LVEF < 30%
Age (y)	32.6 ± 10.8	34 ± 9.39	38.8 ± 7.87	41.13 ± 11.83
Men/women (*n*)	5/1	8/2	12/3	11/4
NYHA class	—	1.22 ± 0.44	1.6 ± 0.57	2.10 ± 0.47
BMI	22.05 ± 0.92	21.7 ± 1.43	26.58 ± 2.89	25.35 ± 3.88
LV EF [%]	63 ± 4	47.77 ± 2.81	35.80 ± 3.42	22.33 ± 4.06
LV EDD [mm]	51.34 ± 3.74	53.44 ± 1.74	65.13 ± 7.38	71.53 ± 6.65
LV ESD [mm]	30 ± 1.4	34.55 ± 4.74	47.73 ± 8.75	60.13 ± 5.46
ESV [mL]	45 ± 8	52 ± 4.2	124.2 ± 44.76	188.93 ± 49.25
EDV [mL]	102 ± 13.2	105 ± 24.8	182.86 ± 57.9	241.8 ± 79.15
NT-proBNP [pg/mL]	64.4 ± 1.4	78.61 ± 1.3	999.8 ± 1625.1	1709.6 ± 2314.3
Creatinine [*µ*mol/L]	68.9 ± 10.2	72.01 ± 10.5	72.22 ± 13.09	76.46 ± 12.5
Glucose [mmol/L]	5 ± 0.40	5.03 ± 0.45	5.02 ± 0.40	5.51 ± 0.91
Cholesterol [mmol/L]	3.3 ± 1.42	3.33 ± 1.97	5.13 ± 0.58	4.99 ± 1.55
TG [mmol/L]	1.45 ± 0.21	0.85 ± 0.74	1.62 ± 0.77	1.53 ± 0.86
HDL [mmol/L]	1.2 ± 0.81	1.05 ± 0.84	1.39 ± 3.14	1.40 ± 0.56
LDL [mmol/L]	2.12 ± 0.50	2.01 ± 1.39	3.14 ± 0.60	2.45 ± 1.34
Diuretics	—	1	8	14
Digitalis	—	1	4	9
ACE inhibitors	—	2	3	3
*β*-blockers	—	3	6	5

**Table 2:** Primers used for quantitative real-time polymerase chain reaction.

Gene	Primer sequence
PPAR*α*	F: 5′-AGA-TTT-CGC-AAT-CCA-TCG-GC-3′
	R: 5′-GCG-TGG-ACT-CCG-TAA-TGA-TA-3′

FAT/CD36	F: 5′-GGA-AAG-TCA-CTG-CGA-CAT-GA-3′
	R: 5′-CCT-TGG-ATG-GAA-GAA-CGA-ATC-3′

CPT-1	F: 5′-GGT-GAA-CAG-CAA-CTA-TTA-TGT-C-3′
	R: 5′-ATC-CTC-TGG-AAG-TGC-ATC-3′

GLUT4	F: 5′-GCT-ACC-TCT-ACA-TCA-TCC-AGA-ATC-TC-3′
	R: 5′-CCA-GAA-ACA-TCG-GCC-CA-3′

PDK-4	F: 5′-TAC-TCC-ACT-GCA-CCA-ACG-C-3′
	R: 5′-AAT-TGG-CAA-GCC-GTA-ACC-A-3′

Beclin-1	F: 5′-TGG-ATC-ACC-CAC-TCT-GTG-AG-3′
	R: 5′-TTA-TTG-GCC-AGA-GCA-TGG-AG-3′

Caspase-3	F: 5′-AGA-ACT-GGA-CTG-TGG-CAT-TGA-G-3′
	R: 5′-GCA-TTG-TCG-GCA-TAC-TGT-TTC-AG-3′

GAPDH	F: 5′-GAA-GTA-GGT-GAT-GGG-ATT-TC-3′
	R: 5′-CAA-GCT-TCC-CGT-TCT-CAG-CC-3′

*β*-actin	F: 5′-TGC-CAT-CCT-AAA-AGC-CAC-3′
	R: 5′-TCA-ACT-GGT-CTC-AAG-TCA-GTG-3′

**Table 3:** Morphometric data.

	Control (*n* = 6) LVEF > 60%	Group (I) (*n* = 10) LVEF 50–45%	Group (II) (*n* = 15) LVEF 41–30%	Group (III) (*n* = 15) LVEF < 30%
Myocyte cross-sectional area [*μ*m^2^]	415 ± 48	452 ± 83	548 ± 94	526 ± 127
Myocyte longitudinal area [*µ*m^2^]	1294 ± 146	1642 ± 198^*∗*^	2094 ± 458^*∗*^	2298 ± 477^*∗*#^
Myocyte degeneration [%]	0.28 ± 0.1	1.25 ± 0.9	6.72 ± 3.5	10.23 ± 4.6
Desmin dominated pattern	I	IIA	IIB	III
Myocyte death				
Apoptotic [‰]	0	0.03 ± 0.004	0.05 ± 0.008	0.02 ± 0.001
Autophagic [‰]	0.4 ± 0.02	1.01 ± 0.8	3.5 ± 2.18	7.92 ± 2.39
Glycogen storage (final score)	Normal	Low	Severe	Moderate
Ultrastructural pathology (final score)	No	Mild	Moderate	Severe

^*∗*^ *p* < 0.05 versus control group, ^#^ *p* < 0.05 versus Group (I).
